# The Relationship Between Bullying Victimization and Malevolent Creativity in Chinese Middle School Students: A Moderated Chain Mediation Model

**DOI:** 10.3390/bs15101386

**Published:** 2025-10-13

**Authors:** Tiancheng Li, Jiantao Han, Zhendong Wan, Xiaohan Pan, Ruoxi Li, Chunyan Yao

**Affiliations:** School of Educational Science, Anhui Normal University, Wuhu 241000, China; 13505601221@163.com (T.L.); shorhaner@163.com (X.P.); 13085531315@163.com (R.L.); 15855129662@163.com (C.Y.)

**Keywords:** bullying victimization, malevolent creativity, trait anger, social mindfulness, emotion regulation

## Abstract

**Background**: Bullying victimization is a common phenomenon that can affect middle school students’ malevolent creativity. However, the underlying mechanisms between the two remain unclear. This study integrates the social hostility model and the Conservation of Resources theory to further explore the relationship between bullying victimization and malevolent creativity, the mediating roles of trait anger and social mindfulness, and the moderating role of emotion regulation, thereby advancing the research and filling the relevant gaps. **Method:** Using validated Chinese versions of the Olweus Bullying Scale, Trait Anger Scale, Social Mindfulness Self-Report Scale, malevolent Creativity Behavior Scale, and Emotion Regulation Questionnaire, *N* = 860 students were surveyed in a cross-sectional design. **Results:** The results showed that bullying victimization was positively related to malevolent creativity (total effect size β = 0.44), with a direct effect of size β = 0.17 and significant indirect effects via social mindfulness (β = 0.05; 11%), trait anger (β = 0.18; 41%), and the sequential path (β= 0.04; 9%). Emotion regulation moderated the links of social mindfulness and trait anger with malevolent creativity, such that higher emotion regulation strengthened the negative association for social mindfulness and weakened the positive association for trait anger. **Implications:** These findings suggest that school-based programs targeting emotion regulation and social mindfulness, alongside anger management components, may help mitigate the harmful impact of bullying on malevolent creativity.

## 1. Introduction

Creativity, defined as the ability to solve problems uniquely and generate novel, valuable products (Kaufman & Sternberg, 2010), is indispensable in today’s rapidly evolving information era (Yu & Zhang, 2019). Although often associated with positive terms like “talent” and “intelligence” (Harris, 2013), highly creative individuals may exhibit unethical or unpopular deviant behaviors, as hinted by the title of Gao Ming’s book *Genius on the Left, Madman on the Right* (Cropley et al., 2014). In reality, creativity itself is morally neutral (Hao & Yang, 2016); however, when combined with ill intent, it becomes malevolent creativity (Becker, 2014). While meeting the core criteria—“novelty” and “effectiveness” (Sternberg & Lubart, 1996)—malicious creativity inflicts destruction (Cropley et al., 2008; Eisenman, 2008), ranging from large-scale threats like terrorism to everyday harms like deception (Cropley et al., 2014; Gong & Liu, 2016; Hao & Yang, 2016), endangering others’ safety and well-being (Rui et al., 2021; D. Wang et al., 2022). Adolescents, who are in a critical stage of physical and psychological development, face academic pressure and ecological risks like school bullying and family dysfunction (G. Liu, 2024), which may trigger malevolent creative behaviors. These not only disrupt their cognitive development and moral judgment (Kapoor & Kaufman, 2022), degrading their social skills (Perchtold-Stefan et al., 2022), but also harm the mental and physical health of their targets. Thus, studying adolescent malevolent creativity is of urgent social and educational significance.

Previous research has identified multiple external factors influencing adolescent malevolent creativity, including social rejection, parental rejection, and school bullying (Bai & Yang, 2025; W. Yang et al., 2025; Sun et al., 2024). Among these, bullying victimization in schools is an important risk factor predicting adolescent malevolent creativity (W. Yang et al., 2025): evidence shows that higher levels of bullying victimization correlate with increased malevolent creativity (W. Yang et al., 2025). However, school bullying also exerts an indirect influence through adolescents’ internal psychological factors. According to the social hostility model (Van Lange & Van Doesum, 2015), malevolent creativity is shaped by a complex interplay of the social environment, individual cognition, and personality traits, suggesting underlying internal mechanisms (Gong & Liu, 2016) between bullying victimization and malevolent creativity. While some studies have explored the internal mediating mechanism between bullying victimization and adolescent malevolent creativity from the cognitive dimension of anger rumination (W. Yang et al., 2025), few have investigated the combined roles of cognitive factors (e.g., social mindfulness) and personality traits (e.g., trait anger). Additionally, regarding protective factors, prior research has predominantly focused on variables such as moral reasoning (J. Zhao et al., 2022), coping styles, forgiveness, and approach-avoidance motivation (Li et al., 2022, 2024; Sun et al., 2024), overlooking the moderating role of emotional factors (e.g., emotion regulation) in preventing adolescent malevolent creativity. In summary, integrating cognitive, personality, and emotion regulation perspectives offers great potential for understanding the mechanisms linking school bullying victimization and middle school students’ malevolent creativity. Compared with prior studies that typically focused on either cognitive or emotional mechanisms alone, this study simultaneously incorporates cognitive (social mindfulness), personality (trait anger), and emotional (regulation) perspectives. This integrated approach highlights the novelty of our model and clarifies how multiple psychological processes jointly shape malevolent creativity. Therefore, this study addresses a research gap by constructing a moderated mediation model to examine the relationship between bullying victimization and malevolent creativity, the chained mediating roles of social mindfulness and trait anger, and the moderating role of emotion regulation, thereby advancing the field of malevolent creativity research.

### 1.1. Bullying Victimization and Malevolent Creativity

School life plays a pivotal role in adolescent development, and school bullying has lasting negative effects (Shen et al., 2019). Bullying victimization—characterized by being the target of repeated, intentional physical or psychological attacks that exploit power imbalances (Olweus, 1997)—often triggers psychological distress such as elevated negative emotions and depression (Troop-Gordon, 2017). Drawing from the Sensitivity to Mean Intentions (SeMI) model (Gollwitzer & Rothmund, 2009), individuals sensitive to unfairness in their environment tend to focus on harmful information during adverse situations, potentially leading to extreme behaviors. Research indicates that bullying victims are more likely to engage in self-harm, suicide, and other extreme behaviors (R. Yang et al., 2024), and they demonstrate elevated levels of malevolent creativity (Y. Wang et al., 2024; M. Zhao et al., 2022) as a means of protesting perceived injustice. Moreover, increased exposure to school bullying is associated with heightened aggression in adolescents (Zeng et al., 2024), which may contribute to malevolent creativity (Wu et al., 2024b). Longitudinal studies further confirm a significant positive association between bullying victimization and malevolent creativity (W. Yang et al., 2025; Tong et al., 2024). Therefore, this study posits the following hypothesis:

**H1.**  *Bullying victimization positively predicts malevolent creativity among Chinese middle school students.*

### 1.2. The Mediating Role of Social Mindfulness

Bullying victimization not only directly influences malevolent creativity but also indirectly predicts it by shaping internal cognitive factors such as social mindfulness—the tendency to consider others’ needs and desires in decision-making (Van Lange & Van Doesum, 2015). According to the Conservation of Resources theory (Hobfoll et al., 2018), resource loss, especially that of crucial resources like social support, exacerbates cognitive depletion and triggers avoidance behaviors. Adolescents subjected to physical or verbal violence in bullying contexts often experience physical and mental exhaustion, depleting their cognitive resources. Social mindfulness, reliant on cognitive resources (Van Doesum et al., 2013), may decline as a result of such depletion. Therefore, this study hypothesizes that greater bullying victimization may reduce adolescents’ social mindfulness. Social mindfulness also significantly influences malevolent creativity (J. Yang et al., 2021; W. Yang et al., 2025): according to the Social Hostility Model (Van Lange & Van Doesum, 2015), low social mindfulness (i.e., social hostility) diminishes individuals’ concern for others, fostering negative behaviors and emotions that trigger malevolent creativity. Hence, social mindfulness is likely negatively associated with malevolent creativity. Thus, this study assumes that school bullying increases malevolent creativity by decreasing social mindfulness. Accordingly, we propose the following hypothesis:

**H2.**  *Social mindfulness mediates the relationship between bullying victimization and malevolent creativity.*

### 1.3. The Mediating Role of Trait Anger

Bullying victimization may also indirectly predict malevolent creativity through trait anger, a stable personality trait characterized by enduring tendencies in anger frequency, duration, and intensity (Spielberger & Sydeman, 1994; Spielberger et al., 1999). Trait activation theory (Tett & Guterman, 2000; Tett et al., 2013) posits that specific situations can trigger latent traits, driving corresponding behaviors. Adolescents experiencing bullying are highly likely to have their trait anger activated by aggressive acts and verbal violence in hostile environments. Empirical evidence demonstrates that bullying victimization positively correlates with trait anger (Camodeca & Goossens, 2005; Walters & Espelage, 2018; Zahlquist et al., 2023). Thus, higher victimization levels are associated with greater trait anger in adolescents. Moreover, trait anger is a significant risk factor for malevolent creativity: research confirms a positive association between them (Perchtold-Stefan et al., 2021), with adolescents higher in trait anger exhibiting more malevolent creativity (Wu et al., 2024a; Rui et al., 2021). The integrative cognitive model (Wilkowski & Robinson, 2008) further suggests that individuals with high trait anger are prone to interpreting ambiguous situations as hostile while lacking the resources or ability to regulate such hostile cognitive biases, thereby increasing the likelihood of malevolent creativity. Therefore, this study hypothesizes that school bullying elevates adolescents’ malevolent creativity by increasing their trait anger. Accordingly, we propose the following hypothesis:

**H3.**  *Trait anger mediates the relationship between bullying victimization and malevolent creativity in middle school students.*

### 1.4. The Chained Mediating Role of Social Mindfulness and Trait Anger

Given that bullying victimization can indirectly influence malevolent creativity through social mindfulness and trait anger, are these two factors interconnected? Research indicates that low social mindfulness positively predicts anger levels (Van Doesum et al., 2016). Van Lange and Van Doesum (2015) posited that low social mindfulness reflects social hostility, which can trigger anger towards others. Consequently, adolescents with chronically low social mindfulness are more likely to experience anger in daily life, leading to high trait anger. This negative association between social mindfulness and trait anger has been validated in competitive contexts such as sports (Allred & Smith, 1991; Van Doesum et al., 2016; Van Lange & Van Doesum, 2015). Therefore, this study proposes that bullying victimization may increase malevolent creativity by first reducing social mindfulness and then elevating trait anger. We propose the following hypothesis:

**H4.**  *Social mindfulness and trait anger jointly act as a chained mediator in the relationship between bullying victimization and malevolent creativity among middle school students.*

### 1.5. The Moderating Role of Emotion Regulation

While the above analysis suggests that bullying victimization can lead to low social mindfulness, high trait anger, and increased malevolent creativity in adolescents, these effects vary across individuals, potentially linked to protective psychological traits such as emotion regulation. Emotion regulation refers to the process by which individuals monitor, evaluate, and modify their emotional responses through certain strategies (Gross, 1999), mainly consisting of cognitive reappraisal and expressive suppression (Gross, 1998). According to Gross’s emotion regulation theory (Gross, 2002; Gross & John, 2003), individuals with strong emotion regulation skills tend to employ cognitive reappraisal, a strategy associated with higher happiness and satisfaction. In contrast, people who often use expressive suppression tend to have lower mental health, which can lead to cognitive resource loss and negative emotions. For example, previous research has shown that adolescents with strong emotion regulation tend to demonstrate high social mindfulness (Slutsky et al., 2017), low trait anger (Szasz et al., 2011), and reduced engagement in immoral behaviors such as antisocial or deviant acts, thereby decreasing malevolent creativity (Helion & Ochsner, 2018). In contrast, individuals with poor emotion regulation exhibit the opposite traits. Additionally, a study on college students revealed that higher emotion regulation weakens the positive association between childhood trauma and malevolent creativity (Qiu et al., 2023). Overall, emotion regulation appears to be a protective factor against adolescent malevolent creativity, potentially moderating the relationship between negative psychological traits and malevolent creativity.

Therefore, this study proposes the following hypotheses:

**H5.**  *Emotion regulation moderates the relationship between social mindfulness and malevolent creativity among middle school students.*

**H6.**  *Emotion regulation moderates the relationship between trait anger and malevolent creativity among middle school students.*

**Figure 1:**
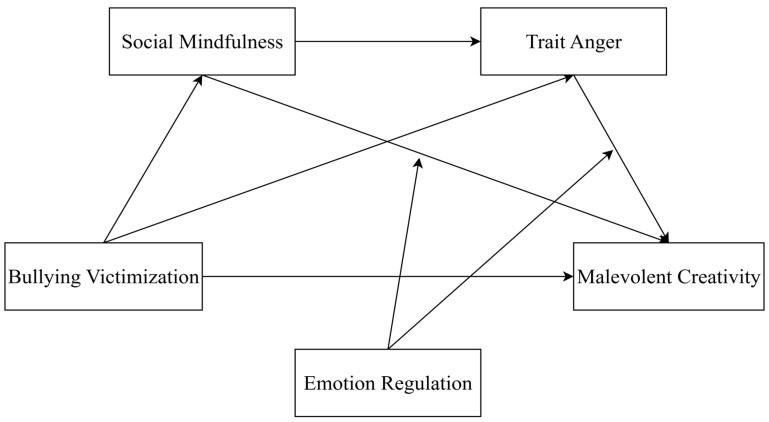
Chain-mediated model of regulation.

In summary, integrating cognitive, personality, and emotion regulation perspectives offers great potential for understanding the mechanisms linking school bullying victimization and middle school students’ malevolent creativity. Unlike earlier research emphasizing anger rumination or justice beliefs in explaining bullying-related outcomes, our moderated chain mediation framework extends this literature by linking social mindfulness, trait anger, and emotion regulation into a single explanatory model. Moreover, unlike prior studies that typically focused on either cognitive or emotional mechanisms alone, this study simultaneously incorporates cognitive (social mindfulness), personality (trait anger), and emotional (regulation) perspectives. This integrated approach highlights the novelty of our model and clarifies how multiple psychological processes jointly shape malevolent creativity. Therefore, this study addresses a research gap by constructing a moderated mediation model (See Figure 1) to examine the relationship between bullying victimization and malevolent creativity, the chained mediating roles of social mindfulness and trait anger, and the moderating role of emotion regulation, thereby advancing the field of malevolent creativity research.

## 2. Materials and Methods

### 2.1. Study Design

This study employed a cross-sectional survey design to investigate the relationships between bullying victimization and malevolent creativity among middle school students and tested a moderated chain mediation model integrating social mindfulness, trait anger, and emotion regulation. Data were collected at a single time point using a structured self-report questionnaire.

### 2.2. Participants and Sampling Procedure

A convenience sampling method was used to recruit participants. We reached out to the school administrators of two middle schools in Wuhu City, Anhui Province, recruited 950 students to participate in a questionnaire survey and distributed the questionnaires during regular class sessions under the supervision of classroom teachers. Participation was voluntary, and written parental/guardian consent was obtained in addition to student assent in accordance with the Declaration of Helsinki and relevant guidelines. All participants were informed of confidentiality protections and their right to withdraw at any time. Data collection took place from March to April 2025. Invalid questionnaires (e.g., incomplete responses, patterned answers, or response times shorter than 2 min) were excluded, leaving a final sample of 860 students (effective response rate: 90.5%). The sample comprised 390 males (45.3%) and 470 females (54.7%), with an average age of 14.83 years (SD = ±1.67); 440 (51.2%) were in junior high and 420 (48.8%) in senior high. The study was reviewed and approved by the Anhui Normal University Ethics Committee (protocol code:AHUN-ET2025005, date of approval 27 February 2025).

### 2.3. Measures

#### 2.3.1. Bullying Victimization

The Chinese version of Olweus’ Bully/Victim Scale (revised by Zhang & Wu, 1999) was adopted (Olweus, 1993), consisting of 6 items, such as “I was threatened or intimidated at school”. Responses used a 5-point scale (“0 = no” to “4 = 5 times or more”), with higher average scores indicating more severe school bullying. The Cronbach’s α of this scale was previously reported to be 0.82 among Chinese middle school students (Da et al., 2018): the internal consistency coefficient for the questionnaire was 0.815 in this study.

#### 2.3.2. Social Mindfulness

The social mindfulness Self-Report Scale developed by Tian et al. (2021) was utilized. Comprising 17 items, it features a second-order four-factor structure, encompassing agreeableness and extraversion traits, such as “I often put myself in others’ shoes” The scale uses a 5-point scoring system, with higher scores signifying a stronger trait-based social mindfulness tendency. In a study on adolescents by Y. Liu (2023), the Cronbach’s α coefficient was 0.89, while in this study, the Cronbach’s α coefficient for this scale was 0.913.

#### 2.3.3. Trait Anger

The Chinese version of Spielberger et al.’s (1999) Trait Anger Scale (revised by Luo) was used (Luo et al., 2011), comprising 10 items such as “When I’m angry, I tend to use foul language.”. Responses used a 4-point scale (“1 = not at all” to “4 = always”), with higher scores reflecting greater trait anger. In a previous study, the Cronbach’s α of the trait anger scale was 0.94 among Chinese middle school students (Zhang et al., 2024): in the present study, the Cronbach’s α of this scale was 0.879.

#### 2.3.4. Malevolent Creativity

The malevolent Creativity Behavior Scale (MCBS) developed by Hao et al. (2016) was employed, consisting of 13 items across three dimensions: hurting others, joking and lying. For example, “I’ve thought of unusual ways to hurt people who stand in the way of my goals”. Responses used a 5-point scale (“1 = not at all” to “5 = always”), with higher scores indicating greater malevolent creativity. The scale previously demonstrated a Cronbach’s α of this scale was 0.94 among Chinese middle school students (Yao et al., 2024) and achieved a Cronbach’s α of 0.934 in this study.

#### 2.3.5. Emotion Regulation

The Emotion Regulation Questionnaire (ERQ; Gross & John, 2003; L. Wang et al., 2007) comprises 10 items across cognitive reappraisal (6 items) and expressive suppression (4 items), rated on a 7-point scale. Consistent with prior work and our hypotheses, the moderation analyses used the cognitive reappraisal subscale as the indicator of emotion regulation (higher scores = greater reappraisal use). The internal consistency was acceptable (α = 0.739) in this sample. For completeness, the subscale reliabilities were α = [fill α = 0.784 for reappraisal], α = [fill α = 0.672 for suppression].

### 2.4. Statistical Analysis

Data analyses were conducted using Mplus 8.0, SPSS 26.0 and the PROCESS macro. Firstly, a common method bias test was conducted using Mplus 8.0 to ensure the absence of significant common method bias issues in this study, which may affect subsequent research. Descriptive statistics and Pearson correlations were secondly calculated. Then, to test mediation effects, PROCESS Model 6 was used and for moderation effects, PROCESS Model 88 was applied. Age, gender, and grade were included as control variables in all models. Finally, significance was evaluated using 5000-sample bias-corrected bootstrapping with 95% confidence intervals. All predictors involved in interaction terms were mean-centered (standardized) prior to computing products.

## 3. Results

### 3.1. Common Method Variance Test

In this study, Harman’s single-factor test was employed to assess common method bias. Factor analysis without rotation was conducted on all items of the five variables. The results revealed that 11 factors had eigenvalues greater than 1, and the first factor explained 22.28% of the total variance. This was below the critical 40%, indicating no severe common method bias in this study. Additionally, a confirmatory factor analysis was performed on the data, setting the common factor at l. The ft indices indicated a poor model fit, as evidenced by *χ*^2^/*df* = 10.42, TLI = 0.38, CFI = 0.40, SRMR = 0.12 and RMSEA = 0.11, Thus, this study did not exhibit a significant issue of common method bias.

### 3.2. Descriptive Statistics and Correlations

Pearson correlation analysis was performed among bullying victimization, malevolent creativity, social mindfulness, trait anger, and emotion regulation. As presented in Table 1, bullying victimization was positively associated with malevolent creativity. Social mindfulness exhibited negative correlations with bullying victimization, malevolent creativity, and trait anger but a positive correlation with emotion regulation. Trait anger was positively associated with both bullying victimization and malevolent creativity. Emotion regulation showed negative correlations with malevolent creativity and trait anger. Overall, the correlations ranged from weak to moderate, suggesting consistent but not overly strong associations among the study variables.

### 3.3. Chain Mediation Effect Test

**Figure 2:**
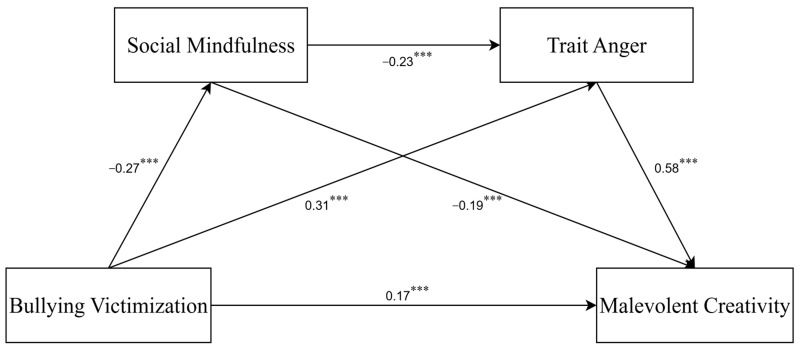
Chain-mediation model with standardized path coefficients (N = 860). *** *p* < 0.001.

Firstly, all variables were standardized. Model 6 of the SPSS Process macro was used to test the mediating role of social mindfulness and trait anger between bullying victimization and malevolent creativity, as shown in Table 2. After age, gender, and grade were controlled for, the direct effect size of bullying victimization on malevolent creativity was 0.17 (95% *CI* [0.08, 0.26]), and the R^2^ value for malevolent creativity was 0.32. Social mindfulness exhibited a mediating effect size of 0.05 (95% *CI* [0.03, 0.08], accounting for 11.36% of the total effect), and its R^2^ value was 0.04. Trait anger had a stronger mediating effect size of 0.18 (95% *CI* [0.13, 0.23], or 40.91% of the total effect), and its R^2^ value was 0.19. The chained mediating effect of social mindfulness and trait anger was 0.04 (95% *CI* [0.02, 0.06], or 9.09% of the total effect), as presented in Table 3 and Figure 2. Comparatively, trait anger accounted for the largest proportion of the indirect effect (40.91%), followed by social mindfulness (11.36%) and the sequential path (9.09%), highlighting trait anger as the strongest mediator in the model. These results support H2 (SM as a mediator), H3 (trait anger as a mediator), and H4 (sequential SM → TA mediation).

### 3.4. Test of the Moderating Effect of Emotion Regulation

**Figure 3:**
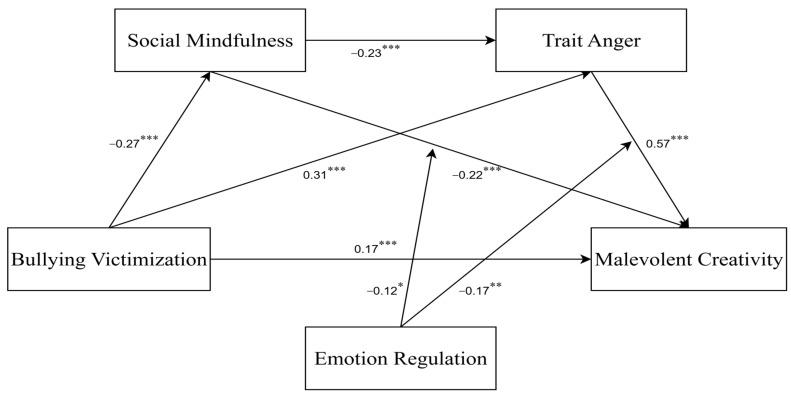
Final moderated chain-mediation model with standardized path coefficients (N = 860). * *p* < 0.05, ** *p* < 0.01, *** *p* < 0.001.

**Figure 4:**
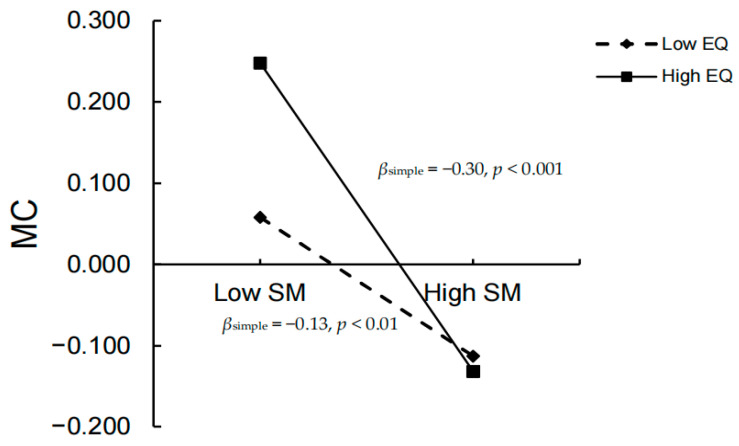
Effect of emotion regulation on the relationship between social mindfulness and malevolent creativity (N = 860, simple slopes at M ± 1SD).

**Figure 5:**
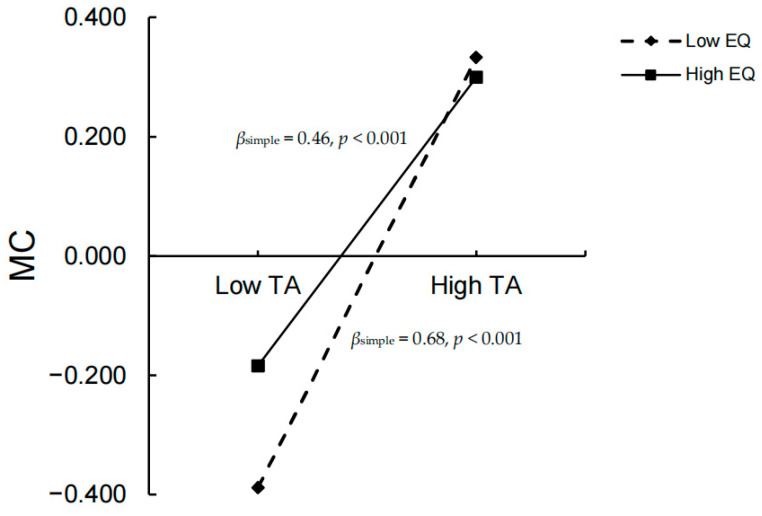
Effect of emotion regulation on the relationship between trait anger and malevolent creativity (N = 860, simple slopes at M ± 1SD).

After the moderator variable was standardized, Model 88 of the SPSS PROCESS macro was used to test the moderating effect of emotion regulation, with the results presented in Table 4. Including the emotion regulation variable in the model revealed that the interaction term of social mindfulness and emotion regulation significantly predicted malevolent creativity (*β* = −0.12, *t* = −2.41, *p* < 0.05, 95% *CI* = [−0.22, −0.02]). Furthermore, the interaction term of trait anger and emotion regulation significantly predicted malevolent creativity (*β* = −0.17, *t* = −3.00, *p* < 0.01, 95% *CI* = [−0.28, −0.06]. These results indicate that emotion regulation moderated both associations. Specifically, Figure 3, Figure 4 and Figure 5 illustrate that high emotion regulation intensified the negative link between social mindfulness and malevolent creativity, while simultaneously weakening the positive link between trait anger and malevolent creativity. Altogether, the model explained 33% of the variance in malevolent creativity (R^2^ = 0.33), demonstrating moderate explanatory power.

The standardized emotion regulation variable was divided into high (*M* + 1*SD*) and low (*M* − 1*SD*) groups. Simple slope analysis showed the following:

Under low emotion regulation (*M* − 1*SD*), the independent mediating effects of social mindfulness and trait anger, as well as their chained mediating effect, were significant: *β*_SM_ = 0.04, 95% *CI* = [0.01, 0.07]; *β*_TA_ = 0.21, 95% *CI* = [0.15, 0.28]; *β*_SM-TA_ = 0.04, 95% *CI* = [0.02, 0.07]. Specifically, social mindfulness significantly negatively predicted malevolent creativity (*β*_simple_ = −0.13, *SE* = 0.05, *t* = −2.82, *p* < 0.01), while trait anger significantly positively predicted it (*β*_simple_ = 0.68, *SE* = 0.05, *t* = 12.54, *p* < 0.001). Under high emotion regulation (*M* + 1*SD*), the independent mediating effect of social mindfulness was strengthened, *β*_SM_ = 0.08, 95% *CI* = [0.04, 0.13], while the effects of trait anger and the chained mediation remained significant but decreased in magnitude, *β*_TA_ = 0.14, 95% *CI* = [0.09, 0.20]; *β*_SM-TA_ = 0.03, 95% *CI* = [0.02, 0.05]. The simple slope analysis showed that as emotion regulation increased, the negative predictive effect of social mindfulness on malevolent creativity intensified (*β*_simple_ = −0.30, *SE* = 0.05, *t* = −5.68, *p* < 0.001), and the positive predictive effect of trait anger weakened (*β*_simple_ = 0.46, *SE* = 0.06, *t* = 7.76, *p* < 0.001).

## 4. Discussion

This study employed a moderated mediating model to investigate the relationship between bullying victimization and malevolent creativity among middle school students. Beyond confirming expected associations, the findings contribute to theory by clarifying how cognitive (social mindfulness), personality (trait anger), and emotional (regulation) factors jointly explain the development of malevolent creativity.

### 4.1. Bullying Victimization and Malevolent Creativity

This study confirms a positive association between school bullying victimization and malevolent creativity in middle school students, supporting Hypothesis 1. These findings align with those of prior studies showing that greater exposure to bullying increases malevolent creativity (Tong et al., 2024). The results also reinforce the Sensitivity to Mean Intentions (SeMI) model (Gollwitzer & Rothmund, 2009), which posits that perceived unfair treatment enhances malevolent creativity, whereas perceived fairness fosters more positive creative behaviors. According to this model, when middle school students experience bullying in threatening environments, highly sensitive individuals are more likely to perceive signals of unfair treatment. This perception of malice can trigger distrust and activate malevolent creativity as a means to restore fairness and protect these individuals’ interests (Clark & James, 1999). In the Chinese school context, where peer pressure and collectivist norms are salient, bullying may be especially likely to trigger perceptions of unfairness, making the link to malevolent creativity particularly pronounced. Although age, gender, and grade were controlled for, exploratory heterogeneity by gender or grade could exist, warranting follow-up tests in future work.

### 4.2. The Mediating Role of Social Mindfulness

This study further found that social mindfulness mediates the relationship between school bullying and malevolent creativity, supporting Hypothesis 2. Middle school students experiencing long-term bullying tend to exhibit low social mindfulness, which in turn increases malevolent creativity. On the one hand, according to the Conservation of Resources theory (Hobfoll et al., 2018), middle school students facing negative experiences like school bullying may deplete their cognitive resources, resulting in a decline in social mindfulness, a process driven by cognitive resources. On the other hand, the social hostility model (Van Lange & Van Doesum, 2015) suggests that low social mindfulness reflects social hostility. For bullied students, low social mindfulness indicates high levels of social hostility, which can trigger negative behaviors and emotions like conflict and hatred, thereby fostering malevolent creativity. However, compared with that of trait anger, the mediating role of social mindfulness was weaker, suggesting that cognitive empathy-related deficits play a role but may be overshadowed by stronger emotional responses. This pattern suggests that cognitive perspective-taking deficits may increase risk, but affective reactivity (anger) plays a comparatively larger role in translating victimization into malevolent creativity.

### 4.3. The Mediating Role of Trait Anger

This study also confirms that trait anger mediates the relationship between bullying victimization and malevolent creativity, supporting Hypothesis 3. Middle school students experiencing long-term bullying tend to exhibit higher levels of trait anger and malevolent creativity. These results align with trait activation theory (Tett & Guterman, 2000; Tett et al., 2013) and the integrative cognitive model (Wilkowski & Robinson, 2008). On the one hand, according to trait activation theory, the “latent” trait anger in individuals can be activated when they are in a negative situation of being bullied, leading to frequent and sustained anger. On the other hand, according to the integrative cognitive model, when adolescents’ trait anger is activated, individuals with high trait anger are more likely to make hostile attributions of bullying-related information, increasing their likelihood of hostile behaviors and thereby enhancing malevolent creativity. The stronger effect of trait anger as compared with social mindfulness implies that interventions focusing on anger management and attribution retraining may yield greater reductions in malevolent creativity than programs targeting empathy alone. Accordingly, attribution-retraining and anger management components may be especially impactful relative to empathy-only trainings.

### 4.4. The Chained Mediating Role of Social Mindfulness and Trait Anger

The results support Hypothesis 4, demonstrating that social mindfulness and trait anger form a chained mediator between school bullying and malevolent creativity. Specifically, bullied adolescents exhibit lower social mindfulness, which in turn elevates trait anger and enhances malevolent creativity. This aligns with Van Lange’s social hostility model (Van Lange & Van Doesum, 2015), which posits that prolonged bullying reduces social mindfulness (W. Yang et al., 2025), making latent trait anger more susceptible to activation (Lee et al., 2020), thereby triggering malevolent creativity. This sequential pathway underscores the interdependence of cognitive and emotional processes, where reduced perspective-taking can escalate anger responses, ultimately fostering destructive forms of creativity.

### 4.5. The Moderating Role of Emotion Regulation

The results confirm Hypotheses 5 and 6, indicating that emotion regulation moderates the associations between social mindfulness/malicious creativity associations. Specifically, middle school students with high emotion regulation are less likely to exhibit low social mindfulness and trait anger activation when facing bullying, thereby inhibiting malevolent creativity. In other words, emotion regulation mitigates the negative effects of bullying-induced low social mindfulness and high trait anger, serving as a protective factor against malevolent creativity. This finding aligns with Gross’s emotion regulation theory (Gross, 2002; Gross & John, 2003), highlighting cognitive reappraisal as a protective mechanism. In practical terms, school-based social emotional learning programs that teach reappraisal and self-regulation strategies could buffer the impact of bullying and reduce the likelihood that victims turn to malevolent creativity as a coping strategy. Practical modules may include brief reappraisal drills (ABC worksheets), anger cue monitoring, and perspective-taking exercises embedded into weekly homeroom periods.

## 5. Strengths and Limitations

By simultaneously examining external, cognitive, and personality factors, together with emotion regulation, this study advances theoretical research by clarifying how multiple psychological processes jointly shape malevolent creativity. This integrated model goes beyond earlier work that typically considered only one or two factors in isolation. For practice, the results suggest that educators should not only aim to reduce bullying and promote harmonious school environments but also implement structured social emotional learning programs to strengthen social mindfulness. Parents can contribute by modeling constructive anger management strategies, while schools may incorporate emotion regulation training (e.g., cognitive reappraisal exercises) into curricula to help students adaptively cope with negative experiences.

This study has several limitations. First, its reliance on self-reported data may have led to social desirability bias. Future research could incorporate experimental methods to obtain more accurate data. Second, the cross-sectional design employed in this study could not fully establish causal relationships due to the lack of temporal precedence. Future research should adopt longitudinal tracking designs or experimental methods to clarify the temporal dynamics and intrinsic mechanisms among the variables. Third, the sample was limited to Anhui Province only, lacking representativeness of other regions or socio-economic levels, which may affect the external validity. This indicates the need for broader sampling. In the future, we will expand the geographical scope and conduct sampling in other provinces to enhance the representativeness of the sample and improve the external validity of the data. Future research could explore the moderating roles of coping styles and approach-avoidance motivation in the mechanisms of malevolent creativity. In addition, socioeconomic status and school type (urban/rural) were not collected, which may have limited generalizability; future studies should include these demographics. Finally, the fact that this study did not involve a pilot program is also an important limitation. In future research, we will conduct pilot studies. Confirmatory factor analyses and distributional diagnostics (e.g., skewness/kurtosis) can also be reported in supplementary materials to further establish construct validity and model assumptions.

## 6. Conclusions

This study investigated the relationships among bullying victimization, social mindfulness, trait anger, emotion regulation, and malevolent creativity. The results support our hypotheses, revealing a positive association between bullying victimization and malevolent creativity in middle school students. Social mindfulness and trait anger act as chained mediators in this relationship. Additionally, emotion regulation moderates the effects of social mindfulness and trait anger on malevolent creativity, serving as a protective factor: higher emotion regulation weakens the link between low social mindfulness and malevolent creativity, as well as that between trait anger and malevolent creativity. These findings suggest that interventions aimed at enhancing social mindfulness and fostering healthy trait anger cognition among middle school students may mitigate the impact of bullying victimization on malevolent creativity.

These findings carry several practical implications. Schools could design prevention programs that combine anti-bullying measures with interventions targeting social mindfulness, anger management, and emotion regulation skills. Policymakers may consider embedding social emotional education into the standard curriculum, while families can reinforce these skills at home. These findings support school-based programs that combine anti-bullying efforts with training in emotion regulation (reappraisal), social mindfulness, and anger management, motivating longitudinal/experimental tests of such programs’ effectiveness.

## Figures and Tables

**Table 1:** Descriptive statistics and correlation between variables (N = 860).

	M	SD	1	2	3	4	5
1. BV	0.23	0.47	1				
2. MC	1.74	0.72	0.27 ***	1			
3. SM	3.66	0.64	−0.20 ***	−0.33 ***	1		
4. TA	1.83	0.53	0.31 ***	0.50 ***	−0.33 ***	1	
5. ER	4.41	0.67	−0.02	−0.10 **	0.37 ***	−0.14 ***	1

Note: *N* = 860; ** *p* < 0.01, *** *p* < 0.001; Bullying Victimization = BV; Malevolent Creativity = MC; Social Mindfulness = SM; Trait Anger = TA; Emotion Regulation = ER.

**Table 2:** Results of the mediating effect of social mindfulness and trait anger (N = 860).

	Model 1 SM			Model 2 TA			Model 3 MC		
	B	SE	t	B	SE	t	B	SE	t
Age	−0.03	0.03	−0.82	−0.02	0.03	−0.76	0.02	0.03	0.52
Gender	−0.02	0.04	−0.48	0.13	0.03	3.84 ***	−0.10	0.04	−2.38 *
Grade	0.07	0.11	0.60	0.00 ^†^	0.09	0.05	0.17	0.11	1.54
BV	−0.27	0.05	−5.79 ***	0.31	0.04	8.35 ***	0.17	0.05	3.62 ***
SM				−0.23	0.03	−8.98 ***	−0.19	0.03	−5.53 ***
TA							0.58	0.04	13.59 ***
R^2^	0.04			0.19			0.32		
F	8.75 ***			40.23 ***			65.97 ***		

Note: *N* = 860; * *p* < 0.05, *** *p* < 0.001; Actual value of ^†^ lies within the interval of 0.004 to 0.005. Bullying Victimization = BV; Malevolent Creativity = MC; Social Mindfulness = SM; Trait Anger = TA.

**Table 3:** Test of the mediating role of social mindfulness and trait anger between bullying victimization and malevolent creativity. (N = 860).

	Effect Size	Boot SE	Boot LLCI	Boot ULCI	Ratio
Indirect effect: BV-SM-MC	0.05	0.01	0.03	0.08	11.36%
Indirect effect: BV-TA-MC	0.18	0.03	0.13	0.23	40.91%
Indirect effect: BV-SM-TA-MC	0.04	0.01	0.02	0.06	9.09%
Total indirect effect	0.27	0.03	0.20	0.33	61.36%
Direct effect	0.17	0.05	0.08	0.26	38.64%
Total effect	0.44	0.05	0.33	0.54	100.00%

Note: *N* = 860; Bullying Victimization = BV; Malevolent Creativity = MC; Social Mindfulness = SM; Trait Anger = TA.

**Table 4:** Results of the Moderating Role Test of Emotion Regulation (N = 860).

Predictor Variable	β	SE	*t*	95% *CI*	
				*LL*	*UL*
Age	0.03	0.03	0.78	−0.04	0.09
Gender	−0.10	0.04	−2.47 *	−0.19	−0.02
Grade	0.14	0.11	1.32	−0.07	0.36
ER	0.06	0.03	1.83	−0.00 ^†^	0.13
SM	−0.21	0.04	−5.92 ***	−0.29	−0.14
SM × ER	−0.12	0.05	−2.41 *	−0.22	−0.02
TA	0.57	0.04	13.36 ***	0.48	0.65
TA × ER	−0.17	0.06	−3.00 **	−0.28	−0.06
R^2^	0.33				
F	45.76 ***				

Note: *N* = 860; * *p* < 0.05, ** *p* < 0.01, *** *p* < 0.001; Actual value of ^†^ lies within the interval of −0.005 to −0.004; Social Mindfulness = SM; Trait Anger = TA; Emotion Regulation = ER.

## Data Availability

The datasets used and/or analyzed during the current study are available from the first author upon reasonable request.

## Abbreviations

The following abbreviations are used in this manuscript:

BV	Bullying Victimization
MC	Malevolent Creativity
TA	Trait Anger
SM	Social Mindfulness
ER	Emotion Regulation
